# Impact of Pre-Extraction Methods on Apple Blossom Microbiome Analysis

**DOI:** 10.3390/microorganisms13040923

**Published:** 2025-04-16

**Authors:** Nikhil N. Patel, Jonathan R. Gaiero, Muhammad Sulman, Paul Moote, Darlene Nesbitt, Antonet M. Svircev, Walid Ellouze

**Affiliations:** 1 Agriculture and Agri-Food Canada, London Research and Development Centre, 4902 Victoria Avenue North, Vineland Station, ON L0R 2E0, Canadaantonet.svircev@agr.gc.ca (A.M.S.); 2 Department of Biological Sciences, Faculty of Mathematics and Science, Brock University, 1812 Sir Isaac Brock Way, St. Catharines, ON L2S 3A1, Canada

**Keywords:** sonication, grinding, lyophilization, peptide nucleic acid (PNA) blockers, DNA recovery, microbial diversity, bacterial 16S, fungal ITS, taxonomic resolution, microbial community composition

## Abstract

This study examines the effect of pre-extraction methods, namely, sonication, grinding, and lyophilization, and the use of peptide nucleic acid (PNA) blockers on the DNA recovery, diversity, and taxonomic resolution of bacterial and fungal communities in apple blossoms. Sonication was the most successful in recovering bacterial 16S and fungal ITS reads across all the collection points and plots. Lyophilization and grinding led to a significant reduction in fungal read counts, while PNA enhanced the recovery of bacterial 16S reads. Sonication improved the efficiency of DNA extraction and yielded greater diversity in the recovered microbial community. Sonicated samples showed greater sensitivity to temporal shifts in microbial community composition. Communities in sonicated samples contained a larger number of bacterial genera, such as *Bacillus*, *Staphylococcus*, and *Erwinia*, and fungal genera, including *Didymellaceae* and *Cladosporium.* In contrast, lyophilization and grinding led to a reduction in detected taxa. The indicator species analysis determined that 35 bacterial and 21 fungal genera were closely related to sonication, whereas no other pre-extraction method had any associated genera. Our findings suggest that sonication is the most appropriate pre-extraction method for analyzing blossom-associated microbiomes, and that the use of PNA blockers can improve the recovery of bacteria and minimize contamination by host DNA.

## 1. Introduction

The flower microbiome consists of a community of microorganisms that inhabit various parts of the blossom [1]. The nutrient-rich stigma and hypanthium provide an environment necessary for plant reproductive success and a resident community of microbes that play a critical role in the health and vitality of the flower [2,3]. Stigma exudates, rich in amino acids and carbohydrates, support a diverse microbiota within the intercellular spaces between papillae [4,5,6,7,8,9]. *Erwinia amylovora* is a Gram-negative bacterium that infects members of the Rosaceae family, which includes valuable economic plants such as apples (*Malus* X *domestica*) and pears (*Pyrus communis*). This disease, commonly named fire blight, results in significant global economic losses [10,11,12,13,14]. *E. amylovora* infects apple blossoms by utilizing nutrient-rich exudates on the stigma surface for growth [11,15]. Under wet or high-humidity conditions, the pathogen migrates to the hypanthium, where it ingresses into the plant via the hypanthium [10,13,16,17]. Inside the plant, the pathogen enters an endophytic stage where it produces exopolysaccharides (EPSs) that facilitate biofilm formation which restricts water and nutrient transport, leading to tissue damage and the eventual death of the tree [10,18,19].

Amplicon sequencing methods have been used to investigate the apple blossom microbiome, and whole metagenomic sequencing has been employed to analyze bacterial and fungal communities from individual samples [2,6,7,20]. Interference from non-target DNA associated with plant tissues presents a significant challenge when amplicon sequencing is used to assess apple blossom bacterial populations. Due to the prokaryotic origins of plant plastids and mitochondria, blossom samples contain 16S rRNA genes, resulting in difficulty distinguishing between plant and bacterial sequences. Similarly, in the eukaryotic domain, the fungal ITS region is also present in plants, leading to off-target amplification. This issue can be mitigated by using highly specific primers that exclude host DNA from amplification [21,22,23]. Unfortunately, plant-related amplicons can still obscure the detection of rare microbial species, leading to the underestimation of microbial diversity and limiting the availability of microbial sequences necessary for downstream analyses [22]. To address this, peptide nucleic acids (PNAs) are used as PCR clamps to selectively bind plant DNA, minimizing off-target amplification [24,25]. PNAs differ from DNA by having a backbone of N-(2-aminoethyl) glycine (AEG) instead of a sugar–phosphate structure [22]. The nucleotides in PNA, attached to the AEG backbone, mimic those in DNA and follow Watson–Crick pairing, forming stable hybrid complexes with plant DNA [15,21]. This binding prevents DNA polymerase from replicating the strand to which it is bound during PCR, thereby preventing off-amplification [23]. The use of PNA clamps can achieve a bacterial sequence recovery rate exceeding 92% in blossom microbiome samples, compared to only 6.4% in samples without PNA clamps [15]. Therefore, PNA has the potential to significantly improve the accuracy and comprehensiveness of microbial community analyses.

To understand the apple blossom microbiome, it is important to document changes in community succession patterns over time and interactions between different species within the community [2,5,6,7,15,26,27,28,29,30,31]. Methodological biases may occur at different phases of microbiome research, beginning with the removal of microbes from plant tissues to methodologies used for DNA extraction [20,32]. Pooling multiple washes has been shown to enhance microbial diversity in the apple fruit carposphere [20]. Similarly, extraction methods such as sonication, oscillation, and centrifugation significantly influence microbial recovery in soil [33]. Liu et al. [33] showed that sonication increased the number of culturable bacteria but reduced alpha diversity with prolonged processing times. Sonication, grinding, and lyophilization are commonly used methods for extracting microorganisms associated with plant tissues and blossoms. Sonication uses an ultrasound bath, where ultrasonic sound waves transfer energy through a medium, typically a buffer, in which the blossoms are submerged [34,35]. The acoustic cavitation generated by these sound waves dislodges epiphytic microorganisms from the surface of the blossoms into the buffer [34,35,36]. In contrast, the grinding method involves macerating blossoms in a buffer, releasing microorganisms from both the surface and internal tissues into the solution [20,37]. Lyophilization, which entails freezing followed by the sublimation of water, is primarily used for long-term microbial preservation [38]. After lyophilization, blossoms are homogenized using a bead-beating technique [39]. This method allows the entire blossom to be processed rather than merely dislodging microorganisms into a buffer. Furthermore, bead beating is especially powerful in destroying the cell walls of hard-to-lyse Gram-positive bacterial and fungal cells, thus freeing their nucleic acids [40,41].

In this study, apple blossoms were collected from two Gala apple orchards: one orchard treated with Blossom Protect™ (active ingredient: *Aureobasidium pullulans*) and streptomycin to control fire blight, and a second orchard treated only with streptomycin. The goal was to evaluate how different pre-extraction methods (sonication, grinding, and lyophilization) influence the detection of microbial communities associated with apple blossoms, with a particular focus on capturing both surface-associated and internal microorganisms such as *Erwinia amylovora*. We selected these methods to represent approaches that might differentially capture surface-attached versus tissue-associated microbes. Specifically, we hypothesized that sonication would primarily recover surface microbes, whereas grinding and lyophilization could also disrupt internal tissues, thereby releasing endophytic organisms. Additionally, this study evaluated whether PNAs are necessary to prevent the off-target amplification of host DNA, or if the extraction method alone is sufficient to minimize host DNA interference.

## 2. Materials and Methods

### 2.1. Orchards, Treatments, and Sampling

This study used two orchards at the Agriculture and Agri-Food Canada farm, located in Jordan Station, ON, Canada, as research orchards. Plot 1 (0.205 hectares; 43.17761° N, 79.36092° W) consisted of Brookfield Gala apple trees on G41 rootstock, planted in 2015. The orchard was arranged in 10 rows of 10 trees/row, with 3.5 m spacing between trees and rows. Guard trees were planted around the perimeter of the orchard. Plot 28 (0.184 hectares; 43.17622° N, 79.36372° W) consisted of Brookfield Gala apple trees, planted in 2015, with trees on either M9 or G41 rootstock. This plot was planted as a high-density orchard using a trellis system, containing 8 rows of 40 trees, with 4.6 m spacing between rows and 1.25 m between trees.

**Figure 1:**
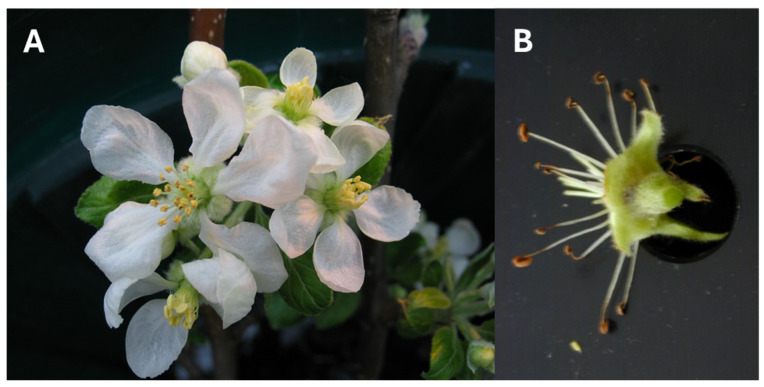
(**A**) Cluster of apple blossoms; (**B**) apple blossom with petals and pedicel removed prior to pre-extraction procedure.

In Plot 1, four blocks were established, each consisting of four trees, with 40 blossoms (10 per tree) collected per block. In Plot 28, four blocks of 5 trees were selected from the G41 rootstock, with 20 blossoms collected per block. Blossoms from Plot 1 were sampled at 30–40% open bloom (T1) and 80–100% open bloom (T2) following streptomycin application. Blossoms from Plot 28 were sampled at 30–40% open bloom (T1) and at petal fall (T3), 7 days after a single streptomycin application. To prevent interference from petal-derived compounds that inhibit PCR reactions, petals and pedicel were removed (Figure 1) before placing the blossoms into sterile 50 mL Falcon tubes (Corning Inc., Corning, NY, USA). Samples were kept on ice during collection and stored at −20 °C until processing.

**Figure 2:**
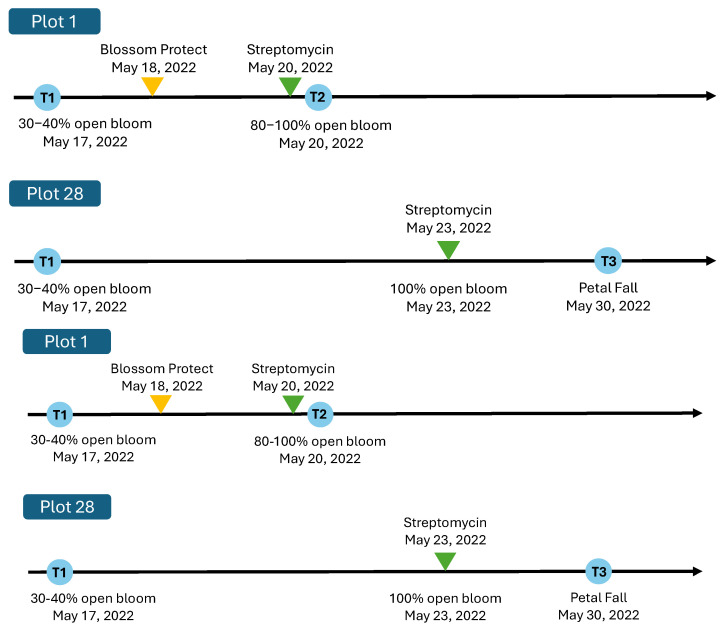
Sampling timeline and fire blight management treatments in apple orchards: Plot 1 and Plot 28.

The two apple orchards followed the same control program, as detailed in Table S1, prior to the first sample collection. Fire blight management in Plot 1 (Figure 2) included a single application of Blossom Protect™ (*Aureobasidium pullulans*; Nufarm, Calgary, AB, Canada) one day after the first collection time (T1), followed by one application of 100 ppm streptomycin 17 (Loveland Products Canada Inc., Dorchester, ON, Canada) just before the second collection time (T2). In Plot 28 (Figure 2), only one streptomycin application was conducted at 100% open bloom.

### 2.2. Processing of Blossoms

Blossom microbiotas were extracted by lyophilization, grinding, and sonication. Six blossoms/block were randomly collected and processed using each of the described treatments, with 4 replicates per treatment (*n* = 4). The DNA extraction procedure started immediately after the pre-extraction step to maintain uniformity between experimental replicates. DNA concentration and purity from all extractions were determined using a Nanodrop microvolume spectrophotometer (Thermo Fisher Scientific Inc., Waltham, MA, USA).

#### 2.2.1. Lyophilization

Blossoms were placed in sterile 50 mL Falcon tubes, covered with sterile cheesecloth, and placed in a Labconco FreeZone 4.5 L −50 °C bench-top freeze dryer (Labconco, Kansas City, MO, USA) for 24–72 h. Once the plant tissue was dry, 5/32” metal beads were added to each tube, and the samples were homogenized in a GenoGrinder (Antylia Scientific, Vernon Hills, IL, USA) for 10 min at 1300 revolutions per minute. DNA was extracted from 20 mg of the ground sample using a DNeasy^®^ PowerSoil^®^ Pro Kit (Qiagen, Germantown, MD, USA, cat. #47016) according to the manufacturer’s protocol.

#### 2.2.2. Grinding

Blossoms were placed in BioReba extraction bags (Bioreba AG, Reinach, Switzerland), to which 15 mL of phosphate buffer (K_2_HP0_4_ at 2.5 g/L and KH_2_PO_4_ at 1.2 g/L, 0.025 M, pH 7.2) was added, and ground using a BioReba AG grinder. After grinding, 10 mL of phosphate buffer was added to the sample. Thirteen ml of the ground slurry was removed and centrifuged at 2205× *g* for 30 min at 4 °C using a Sorvall Lynx 6000 centrifuge (Thermo Fisher Scientific Inc., Waltham, MA, USA). The pellet was resuspended in 1.5 mL of supernatant, and 1 mL was used for DNA extraction with the DNeasy^®^ PowerSoil^®^ Pro Kit (Qiagen, Germantown, MD, USA, cat. #47016) according to the manufacturer’s instructions.

#### 2.2.3. Sonication

Blossoms were placed in 4 oz sterile Whirl-Pak bags (Nasco, Madison, WI, USA), to which 15 mL of phosphate buffer (as above) was added. The bags were sonicated in a Branson 2800 sonicator (Branson Ultrasonics, Brookfield, WI, USA) for 6 min. The suspension was transferred to a 15 mL Falcon tube and centrifuged at 2205× *g* for 30 min, and the pellet was resuspended in 1.5 mL of phosphate buffer. One ml of this sample was used for DNA extraction with the DNeasy^®^ PowerSoil^®^ Pro Kit (Qiagen, Germantown, MD, USA, cat. #47016) according to the manufacturer’s instructions.

### 2.3. DNA Sequencing and Bioinformatics

High-throughput sequencing was conducted by Génome Québec (McGill University, Montreal, QC, Canada) using an Illumina MiSeq platform (2 × 250 bp). The V3-V4 region of the 16S rRNA gene was targeted with primers 341F (5′-CCTACGGGNGGCWGCAG-3′) and 805R (5′-GACTACHVGGGTATCTAATCC-3′) [42], while the V4 region was targeted using primers 515F (5′-GTGCCAGCMGCCGCGGTAA-3′) and 806R (5′-GGACTACHVGGGTWTCTAAT-3′) [43]. PCR amplification of 16S rRNA included the use of PNA clamps, namely, pPNA and mPNA, to block plastid and mitochondrial DNA, respectively, and to selectively amplify bacterial DNA, as previously described for apple blossoms [5,15,44]. A control without PNA clamps was established to evaluate the impact of PNA clamps across extraction methods. The fungal ITS2 region was amplified using ITS86f (5′-GTGARTCATCGARTCTTTGAA-3′) and ITS4R (5′-TCCTCCGCTTATTGATATGC-3′) [45].

Bioinformatic analyses were performed using the QIIME 2 (version 2023.9) wrapper [46]. Adapters, barcodes, and primers were removed with *Cutadapt* [47], and untrimmed reads were discarded. For 16S rRNA, the plugin for DADA2 was used for denoising, dereplication, chimera filtering, and merging paired-end reads [48]. ITS sequences were trimmed using q2-itsxpress (ITSxpress 2.0) due to their variable lengths [49]. This tool identified and trimmed the conserved regions flanking the ITS2 region to improve taxonomic classification accuracy [50].

Following quality filtering and trimming, a total of 924,123 reads and 3695 amplicon sequence variants (ASVs) were obtained for 16S rRNA, while 408,249 reads and 311 ASVs were obtained for ITS. Phylogenetic trees were constructed for 16S rRNA data using the q2-phylogeny plugin (*align-to-tree-mafft-fasttree*) [51,52]. Phylogenetic analyses were not conducted on ITS sequences due to their high variability, which makes them less reliable for such analyses.

The taxonomic classification of bacteria was performed using the classify-sklearn naïve Bayes classifier in q2-feature-classifier [53], with taxonomic classifiers trained against Silva v. 138 reference sequences (99% OTUs) [54]. Reads assigned to plant host DNA, including chloroplasts and mitochondria, were removed. Fungal sequences were classified using UNITE v. 9 reference sequences with dynamic clustering thresholds [55]. Blast+ (classify-consensus-blast) was employed for fungal identification since classify-sklearn and vsearch were unable to accurately classify apple (*Malus* sp.) reads.

### 2.4. Data Analysis

Community analysis was conducted using RStudio (R 4.3.1) and QIIME2, with plots generated in R using the ‘ggplot2’ package [56]. QIIME2 artifacts were imported into R with the ‘qiime2r’ package (https://github.com/jbisanz/qiime2R, accessed on 1 April 2024). Prior to alpha and beta diversity analyses, samples were rarefied to an even number of reads per sample.

Alpha diversity indices, including Shannon diversity and Faith’s phylogenetic diversity, were analyzed using ANOVA with generalized least squares (GLS) models, while observed feature counts (ASV richness) were assessed using a negative binomial distribution. Model residuals were tested, and variance structures were applied if necessary to meet the assumptions of normality and homogeneity of variance.

The beta diversity of bacterial communities was evaluated using the weighted UniFrac phylogenetic distance metric [57], while fungal communities were analyzed with the Jaccard index (presence/absence). Microbial community composition was visualized using a principal coordinate analysis (PCoA) of the dissimilarity matrices. Community differences were tested using permutational multivariate ANOVA (PERMANOVA) and beta-dispersion, implemented with the ‘adonis2’ and ‘betadisper’ functions, respectively, in the ‘vegan’ package [58].

Taxonomic changes were examined through indicator species analysis using the ‘indicspecies’ package [59]. Multi-level pattern analysis (multipatt) was employed to identify taxa distribution patterns across extraction methods. Additionally, compositionally aware differential abundance testing was performed using Analysis of Compositions of Microbiomes with Bias Correction (ANCOM-BC) in the ‘ancombc’ package [60]. ANCOM-BC analysis was conducted on filtered taxa present in at least 10% of samples, with 100 iterations. Pairwise *p*-values were adjusted using the Holm method.

## 3. Results

### 3.1. Impact of Pre-Extraction Methods and PNA Use on Read Recovery

Bioinformatic analyses identified a substantial proportion of non-target reads, primarily originating from plant mitochondria and chloroplasts, despite the use of PNA blockers (Table 1 and Table S2). Rarefaction thresholds were set at 1000 reads for bacterial 16S and 113 reads for fungal ITS (Figures S1 and S2). After the filtering of non-target reads, many samples processed via lyophilization failed to meet the rarefaction threshold, resulting in significant sample loss.

Samples processed with lyophilization showed the highest loss of bacterial 16S and fungal ITS reads, particularly at T1 and T2 in both Plots 1 and 28 (Table 1). Grinding also resulted in substantial fungal ITS losses at the same time points. In contrast, sonication retained sufficient reads for all samples across time points and plots (Table 1).

Bacterial 16S recovery varied across pre-extraction methods and time points. Sonication consistently produced the highest recovery percentages compared to grinding and lyophilization (Table 1). Later bloom stages showed higher bacterial recovery rates across all methods. At T1_P1 (Time 1, Plot 1), bacterial recovery was 7.7% with sonication, 3.5% with grinding, and 0.5% with lyophilization. At T2_P1, recovery increased to 8.2% with sonication, 4.6% with grinding (a 1.3-fold increase over T1_P1), and 1.4% with lyophilization (a nearly 3-fold increase over T1_P1). At T1_P28, sonication resulted in bacterial recovery rates nearly 8 times higher than grinding and over 20 times higher than lyophilization. At T3_P28, sonication yielded more than double the recovery of grinding and over four times that of lyophilization (Table 1). Samples processed with PNA clamps showed significantly higher bacterial recovery rates than those without PNA (Table 1 and Table S2). In the absence of PNA, bacterial 16S reads targeting the V3-V4 or V4 regions were negligible (~0%, Table S2). The highest recovery (36%) was observed at T3_P28 with sonication.

Fungal ITS recovery varied by pre-extraction method and time point. At T1_P1 and T2_P1, fungal recovery showed no difference between grinding and lyophilization. However, sonication increased fungal recovery from 0.32% at T1_P1 to 5.3% at T2_P1 (Table 1). At T1_P28, fungal recovery with sonication reached 0.67%, compared to 0.009% with grinding, while lyophilization did not yield fungal reads. At T3_P28, fungal recovery was highest, allowing a direct comparison of all three methods. Sonication yielded 38 times more fungal reads than grinding and 241 times more than lyophilization (Table 1).

Overall, sonication consistently produced the highest recovery rates for both bacterial 16S and fungal ITS reads across all time points and sample plots. Grinding showed moderate recovery rates, while lyophilization resulted in the lowest recovery and substantial sample loss.

### 3.2. Effect of Pre-Extraction Methods on Alpha and Beta Diversity Across Time Points

Alpha diversity, reflecting species richness and evenness within a sample, was evaluated using Shannon entropy, Faith’s phylogenetic diversity (Faith’s PD), and observed features. Shannon entropy considers both richness and relative abundance, while Pielou’s evenness measures species distribution uniformity. Faith’s PD incorporates evolutionary relationships to assess phylogenetic diversity, and observed features count the number of unique features (frequency > 0) in a sample without considering abundance.

**Figure 3:**
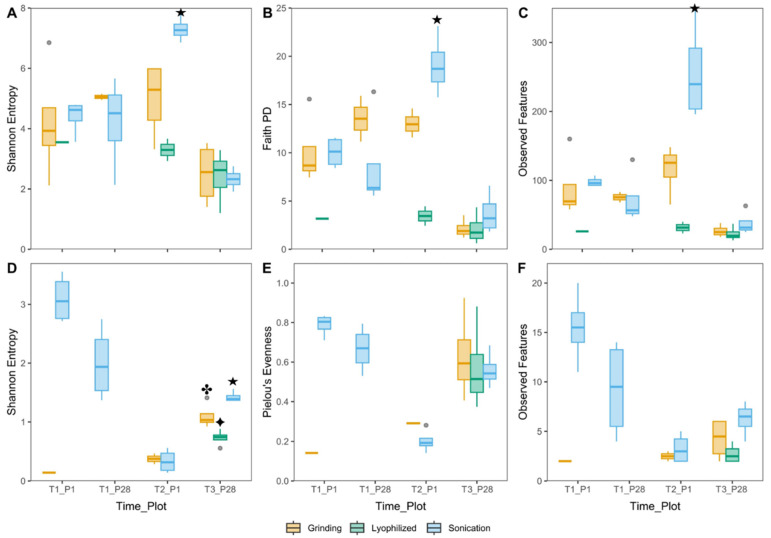
Boxplots of alpha diversity indices for bacterial 16S rRNA (**A**–**C**) and fungal ITS (**D**–**F**) across pre-extraction methods. Alpha diversity indices include Shannon entropy (**A**,**D**), Faith’s phylogenetic diversity (**B**), Pielou’s evenness (**E**), and observed features (**C**,**F**). Statistical analyses were performed using a GLS model for Shannon entropy, Faith’s PD, and Pielou’s evenness, incorporating treatment and time plot as variables. Observed features were analyzed using a negative binomial distribution. Symbols indicate significant differences: (**★**) sonication vs. grinding; (✤) grinding vs. lyophilization; (✦) sonication vs. lyophilization.

In Figure 3A–C, for bacterial 16S rRNA, significant differences can be observed at T2_P1 between sonication and grinding for Shannon entropy (*p* = 0.013), Faith’s PD (*p* = 0.002), and observed features (*p* < 0.001). In Figure 3D, for fungal ITS, at T3_P28, Shannon entropy reveals significant differences between grinding and sonication (*p* = 0.037), grinding and lyophilization (*p* = 0.021), and lyophilization and sonication (*p* = 0.001).

In Plot 1, bacterial diversity increased from T1 to T2, with sonication showing a greater increase compared to grinding. In Plot 28, alpha diversity decreased from T1 to T3 across all pre-extraction methods (Figure 3A–C). Fungal diversity declined from T1 to T2 in Plot 1 and from T1 to T3 in Plot 28 (Figure 3D–F). Despite the overall decline in diversity over time, sonication consistently yielded a higher alpha diversity compared to grinding or lyophilization at most time points.

**Figure 4:**
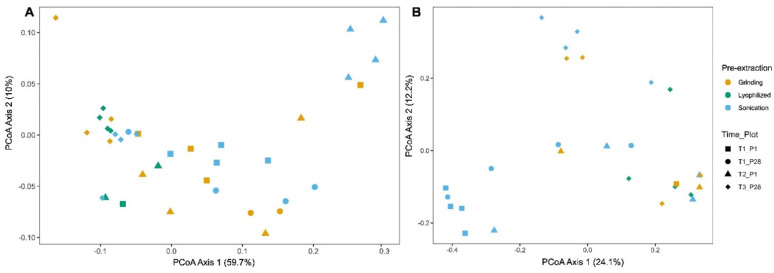
Principal coordinate analysis (PCoA) ordination plots illustrating beta diversity differences between pre-extraction methods. (**A**) Weighted UniFrac dissimilarity for 16S rRNA and (**B**) Jaccard index for ITS.

Beta diversity was used to assess differences in species composition between pre-extraction methods. The PCoA ordination plot for bacterial 16S rRNA shows that the first and second axes explained 59.7% and 10.0% of the variation, respectively (Figure 4A). PERMANOVA analysis revealed significant differences in species composition between sonication and grinding at T2_P1 (*p* = 0.039).

The PCoA ordination plot for fungal ITS shows that the first and second axes explained 24.1% and 12.2% of the variation, respectively (Figure 4B). Time period T3_P28 PERMANOVA analysis identified significant differences between grinding and lyophilization (*p* = 0.029) and between grinding and sonication (*p* = 0.027), but no significant difference between lyophilization and sonication (*p* = 0.116). The distinct clustering of samples within the sonication method, with samples from different time points forming separate clusters, indicated its sensitivity to temporal changes in community composition.

### 3.3. Taxonomic Analyses and Differential Abundance Testing of Bacterial and Fungal Genera

**Figure 5:**
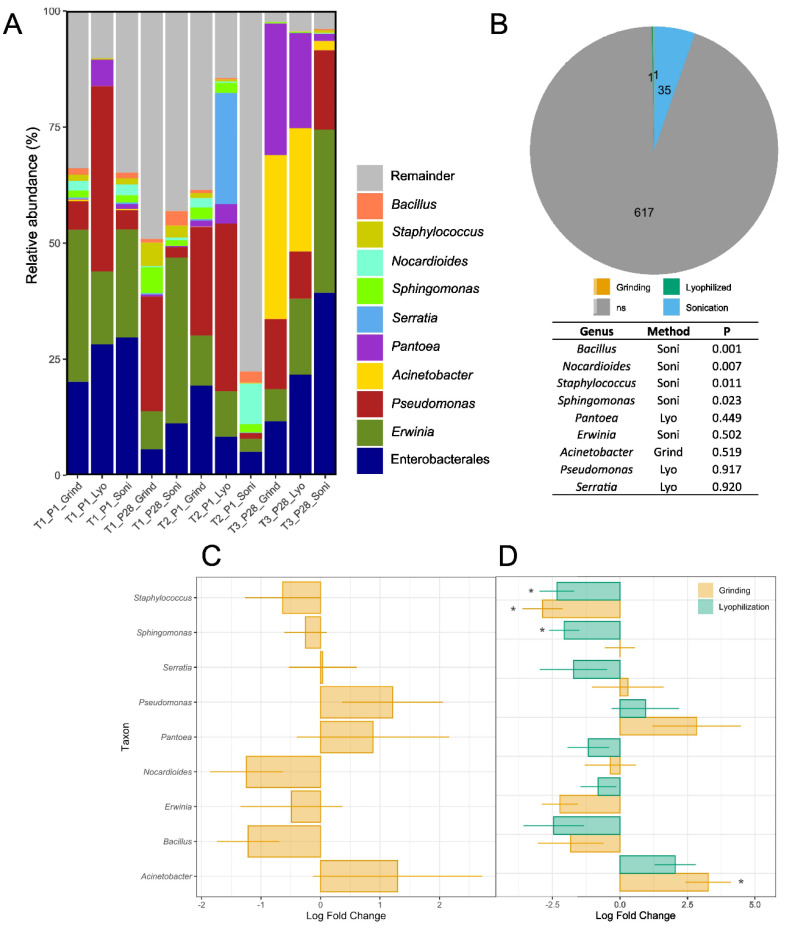
Taxonomic analyses of bacterial 16S rRNA across all time points and pre-extraction methods. (**A**) Relative abundance (%) of the top nine most abundant genera, along with one cluster representing the order Enterobacterales. The remaining reads are grouped and labeled as “Remainder”. (**B**) Indicator species analysis (Indicspecies) showing the number of genera significantly associated with each pre-extraction method. *ns* indicates genera that showed no significant association with any pre-extraction method. The table highlights the nine most abundant genera and their associated *p*-values from the significance test. (**C**) Analysis of Composition with Bias Correction (ANCOM-BC) comparing grinding to sonication (intercept) across all time points, excluding T1-P28. (**D**) ANCOM-BC results at the T3_P28 time point, illustrating significant differences among grinding, lyophilization, and sonication (intercept). Asterisks (*) indicate the level of significance for the ANCOM-BC analysis (* *p* ≤ 0.05).

The taxonomic analysis of bacterial 16S rRNA sequences was conducted to evaluate the impact of pre-extraction methods on the identification and relative abundance of dominant genera. The nine most abundant genera identified were *Bacillus*, *Staphylococcus*, *Nocardioides*, *Sphingomonas*, *Serratia*, *Pantoea*, *Acinetobacter*, *Pseudomonas*, and *Erwinia* (Figure 5A). A notable reduction in the relative abundance of *Erwinia* was observed from T1 to T2 in Plot 1, which had received streptomycin treatments 5 h before blossom collection at T2. The decrease in *Erwinia* abundance was 20.5% using the sonication method and 22.0% using the grinding method (Figure 5A). In contrast, Plot 28, which received a streptomycin application at 100% open bloom 7 days prior to blossom collection at T3, exhibited a slight increase in *Erwinia* abundance from T1 (25.7%) to T3 (35.15%). The abundance of *Erwinia* in Plot 28 remained relatively stable with the grinding method, with 8.2% at T1 and 6.9% at T3 (Figure 5A).

Indicator species analysis and differential abundance testing were conducted to identify bacterial and fungal genera that significantly differ in the abundance (ANCOM-BC) (Figure 5C,D) or fidelity (5B) of taxa between the pre-extraction methods. The results revealed that 35 bacterial genera were associated with the sonication pre-extraction method, while 617 genera showed no association with any of the methods. ANCOM-BC analysis of the nine most abundant genera showed no significant differences or biases between the grinding and sonication methods when all time points were included (Figure 5C). However, an analysis of data from only the T3_P28 time point revealed significant differences between the pre-extraction methods (Figure 5D). Lyophilization showed significantly lower levels of *Staphylococcus* (*p* = 0.030) and *Sphingomonas* (*p* = 0.024) compared to sonication, while the grinding method exhibited lower levels of *Staphylococcus* (*p* = 0.011) but higher levels of *Acinetobacter* (*p* = 0.012) relative to sonication (Figure 5D).

**Figure 6:**
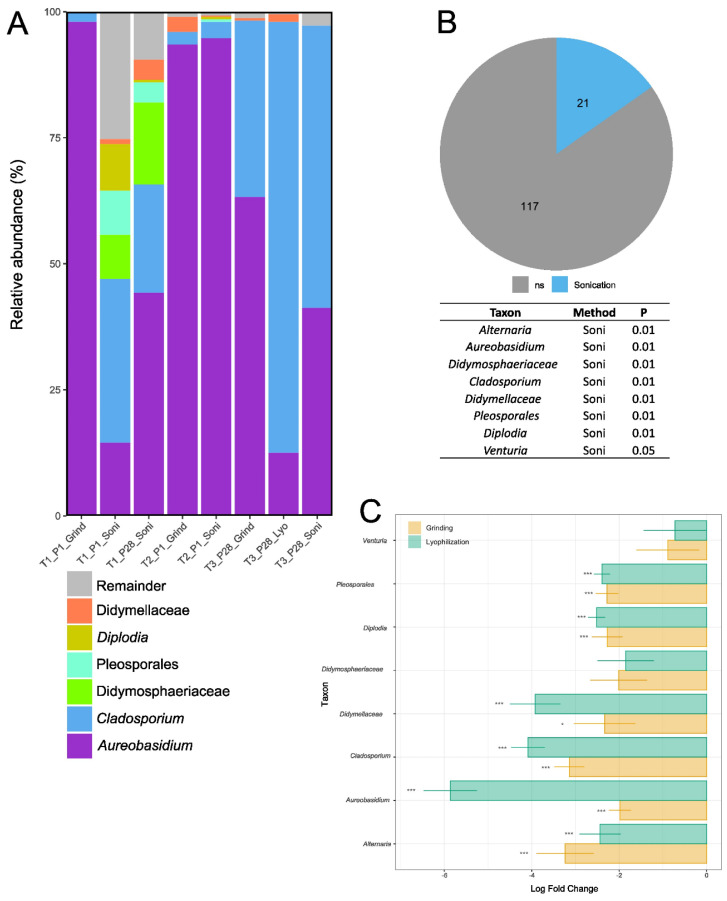
Taxonomic analyses of fungal ITS across all time points and pre-extraction methods. (**A**) Relative abundance (%) of the most abundant taxa. Remaining reads are grouped and labeled as “Remainder”. (**B**) Indicator species analysis showing the number of genera significantly associated with each pre-extraction method at T3_P28 (petal fall). *ns* indicates genera with no significant association with any pre-extraction method. The table highlights the six most abundant taxa and their associated *p*-values from the significance test. (**C**) Analysis of Composition with Bias Correction (ANCOM-BC) at T3_P28, showing significant differences compared to the intercept (sonication). Asterisks (*) indicate the level of significance for the ANCOM-BC analysis (* *p* ≤ 0.05;*** *p* ≤ 0.001).

The taxonomic analysis of fungal ITS sequences was performed to assess the impact of pre-extraction methods on the identification and relative abundance of dominant genera. The six most abundant taxa identified were *Didymellaceae*, *Diplodia*, *Pleosporales*, *Didymosphaeriaceae*, *Cladosporium*, and *Aureobasidium* (Figure 6A). Indicator species analysis at T3_P28, the only time point with sufficient data for a comparison of all three pre-extraction methods, revealed that 21 out of 138 fungal genera were specifically associated with the sonication method (Figure 6B). These included all six dominant taxa, along with Venturia and Alternaria. The remaining 117 genera showed no significant association with any specific pre-extraction method (Figure 6B). ANCOM-BC analysis further evaluated the abundances of the six dominant genera, as well as *Venturia* and *Alternaria*, across the three pre-extraction methods at T3_P28. Lyophilization and grinding yielded significantly lower abundances for all six dominant genera, including *Alternaria* (Figure 6C). *Venturia* exhibited no significant differences between the methods, while *Didymosphaeriaceae* was significantly reduced only in the lyophilization method (Figure 6C).

## 4. Discussion

This study demonstrates the importance of the pre-extraction method and use of PNA to optimize microbial DNA recovery, diversity, and taxonomic resolution in apple blossom microbiome studies. The use of PNA to block the amplification of plant mitochondrial and chloroplast DNA substantially increased bacterial 16S recovery rates, aligning with previous microbiome studies on apple blossoms, where PNA clamps increased bacterial recovery to over 92% [15]. When PNA was not used, bacterial recovery from both the V3-V4 and V4 regions was negligible (~0%). Our results indicate that PNA is essential in plant-associated microbiome studies, as plant DNA dominates amplification. In addition, PNA suppresses non-target amplification without introducing biases in community composition, as demonstrated in eukaryotic phytobiome profiling [61]. This makes PNA a powerful, economic tool for profiling plant microbiomes, particularly those with complex, plant-dominated samples such as apple blossoms.

Sonication was the most effective pre-extraction method, consistently yielding higher microbial recovery rates than grinding and lyophilization across all time points and plots. At T1_P1, bacterial recovery with sonication (7.7%) exceeded that with grinding (3.5%) and lyophilization (0.5%). At T3_P28, sonication doubled bacterial recovery compared to grinding and quadrupled it compared to lyophilization. Fungal ITS recovery exhibited substantial variation across pre-extraction methods, with sonication outperforming grinding and lyophilization. The significantly higher recovery rates observed with sonication suggest that this method effectively reduces host DNA contamination. Despite the use of the ITS86f and ITS4R primers, which are designed to discriminate against plant DNA, fungal reads were largely absent in grinding and lyophilization treatments, indicating that excessive host DNA interference remains a challenge in these methods. The pronounced increase in fungal read recovery with sonication indicates that it may be a superior method for enhancing fungal microbiome profiling in plant-associated samples. This is consistent with prior research highlighting the challenges posed by host DNA contamination, which can dominate sequencing data and obscure the true diversity of the fungal community in plant-associated samples [23]. While PNA clamps have been shown to improve fungal DNA detection by reducing host off-target amplification with universal ITS primers, their benefit appears to be limited when highly fungal-specific primers are used [23]. In this study, we used the ITS86f and ITS4R primers, which are considered sufficiently specific to fungal DNA, and therefore did not combine them with PNA clamps. However, future investigations could explore whether using PNA clamps specifically targeting the plant ITS2 region may further enhance fungal DNA recovery, particularly in host-dominated samples where fungal detection remains challenging.

Lyophilization performed poorly, likely due to DNA loss during dehydration and shearing from bead beating [41], which also released host DNA and reduced bacterial recovery (21.9% vs. 95.7% for sonication). Unlike lyophilization, where the entire blossom was processed but only a subset of a blossom was used for DNA extraction, sonication allowed the entire blossom to be extracted in a single process, improving microbial recovery. Grinding had intermediate performance but selectively lost less abundant taxa, especially fungi, due to mechanical stress.

The presence of PCR inhibitors (e.g., polyphenols, flavonoids, and proteins) in flower tissues possibly further impaired grinding and lyophilization, forming complexes with nucleic acids and reducing enzyme efficiency [62,63]. While the Qiagen DNeasy PowerSoil Pro Kit mitigated the inhibitors, excessive tissue disruption in grinding and lyophilization likely overwhelmed its capacity. In contrast, sonication might have introduced fewer inhibitors, since it is a “gentler” method. This may have led to reduced host DNA contamination, allowing it to achieve higher microbial recovery rates. Based on its superior recovery, sonication may be the most suitable method for profiling plant-associated microbiomes, particularly in plant-dominated samples. Further targeted experiments would be valuable to sort out the individual contributions of host DNA overshadowing, PCR inhibitors, primer biases, and differential extraction efficiency to the observed reduction in microbial reads.

The alpha diversity measures, including Shannon entropy, Faith’s PD, and observed features, consistently favored sonication, highlighting its ability to capture richer and more even microbial communities. At T2_P1, bacterial diversity was significantly higher with sonication compared to grinding, as shown by all three metrics. Similarly, at T3_P28, fungal ITS diversity was markedly higher with sonication than with grinding or lyophilization. Indicator species analysis supported this, showing that 35 of the 654 recovered taxa were more closely associated with sonication. Diversity shifts were evident across plots and time points. In Plot 1, bacterial diversity increased from T1 to T2, despite streptomycin application prior to T2 sampling. This increase was most pronounced with sonication, perhaps a reflection of the enhanced microbial activity and diversity during mid-bloom. Alternatively, streptomycin was bactericidal to the dominant genera *Erwinia* and *Pantoea*, allowing the proliferation of the less abundant taxa and elevating alpha diversity. In contrast to Plot 1, alpha diversity declined over time in Plot 28. This may have been caused by bloom senescence at petal fall which shifts microbial dynamics. This pattern aligns with Boutin et al. [64], who reported a gradual decline in alpha diversity in the apple phyllosphere due to the increasing dominance of taxa such as *Pseudomonas*.

Fungal diversity declined over time, with a sharper decrease in Plot 1, likely due to Blossom Protect^TM^ application after T1. The active ingredient of this product is *Aureobasidium pullulans*, which likely dominated and suppressed other fungal species. This observation is consistent with previous studies [64,65]. In contrast, in Plot 28, where Blossom Protect^TM^ was not applied, *A. pullulans* naturally declined from T1 (30–40% bloom) to T3 (petal fall), allowing *Cladosporium* to dominate. At petal fall, Shannon entropy revealed significant differences between the extraction methods, which means that the applied methods selected different species compositions and, therefore, different species richness. Pielou’s evenness index did not change, indicating that while species richness was different with respect to extraction methods, the abundance of species did not change significantly. This stability shows that the methods may not have greatly transformed the dynamics of fungal populations in terms of dominance or rarity, although the number of species identified was different. Furthermore, indicator species analysis showed that 21 taxa, including *Alternaria* and *Venturia*, were more strongly associated with sonication, highlighting its superior ability to recover fungi compared to grinding and lyophilization. This supports the observation that sonication was more efficient in extracting a more diverse set of fungal species, contributing to the observed differences in species richness.

Beta diversity analyses revealed clear differences in microbial community composition between pre-extraction methods. Principal coordinate analysis (PCoA) showed the distinct clustering of sonicated bacterial 16S samples. PERMANOVA confirmed significant compositional differences between sonication and grinding at T2_P1, which may be caused by sonication’s greater sensitivity in detecting temporal compositional shifts. Fungal ITS PCoA showed lower variance explanation but still distinguished sonication from grinding and lyophilization at T3_P28. The distinct clustering observed in sonication samples across time points emphasizes its ability to capture subtle temporal shifts in microbial communities.

In summary, sonication provided the most comprehensive taxonomic profiles for bacterial and fungal communities, recovering dominant genera such as *Bacillus*, *Staphylococcus*, *Sphingomonas*, and *Erwinia*, as well as low-abundance taxa. In contrast, grinding and lyophilization exhibited selective losses; for example, grinding recovered fewer *Staphylococcus* and *Erwinia* communities, and lyophilization failed to detect *Sphingomonas*. Sonication recovered key fungal genera such as *Didymellaceae* and *Cladosporium*, and pathogens such as *Venturia inaequalis* and *Alternaria alternata*, while grinding and lyophilization showed significant losses. This study used four biological replicates per treatment, with each sample comprising a pool of six blossoms. This approach is commonly used in microbiome pilot studies and provides valuable insights into community composition. While this design allows for meaningful comparisons, a larger sample size would enhance statistical power and improve the generalizability of our findings. Future studies incorporating additional replicates or broader sampling across multiple environments would help to further validate these results and capture potential variations in microbial communities.

## 5. Conclusions

This study highlights the importance of pre-extraction methods and the use of PNA in the estimation of the microbial DNA yield, diversity, and resolution in apple blossom microbiome analyses. Our data show that the usage of PNAs drastically improves the sensitivity of the detection of bacterial populations, especially in the presence of a large amount of host DNA. Sonication was determined to be the best pre-extraction method as it provided better microbial recovery compared to grinding and lyophilization at the different time points and plots. Sonication also reduced host DNA and PCR inhibitors, which led to the capture of a more diverse bacterial and fungal community.

The changes in microbial community dynamics were found to be method-dependent, and sonication was able to capture subtle variations in the microbial communities. In comparison, lyophilization and grinding had limited ability to detect some microbial taxa. Sonication provided a good overview of the taxonomic composition of both bacterial and fungal communities. This study therefore concludes that appropriate extraction techniques and tools should be employed to obtain accurate microbial profiles, especially in complex samples such as apple blossoms which are rich in host DNA. While sonication combined with PNA clamps was the most effective approach in this study, which focused on apple blossoms, its effectiveness in other plant tissues or sample types remains to be tested and may require optimization. It is recommended that future work explores the use of PNA clamps in conjunction with specific ITS primers to improve the recovery of fungal DNA, especially in plant microbiome analyses where the presence of host DNA remains a problem.

## Figures and Tables

**Table 1:** Percentage of bacterial 16S rRNA and fungal ITS reads (mean ± standard error, *n* = 4) retained after rarefaction and filtering of non-target reads (primarily plant-derived) for three pre-extraction methods (grinding, lyophilization, and sonication) across two plots (Plot 1 and Plot 28) and three time points (T1, T2, and T3). Values in parentheses (X/Y) indicate the following: X—the number of samples retained after rarefaction and filtering; Y—the total number of samples before rarefaction and filtering. Bacterial 16S rRNA reads targeted the V3-V4 region, using PNA blockers to remove chloroplast and mitochondrial reads. Fungal ITS reads targeted the ITS2 region and were trimmed with ITSxpress 2.0. Note: ‘−’ indicates the absence of initial samples due to unavailability.

		Bacterial 16S rRNA Reads Recovered After Rarefying and Filtering (%)			Fungal ITS Reads Recovered After Rarefying and Filtering (%)		
Method	Orchard	T1	T2	T3	T1	T2	T3
Grinding	Plot 1	3.500 ± 2.200 (4/4)	4.600 ± 1.500 (4/4)	−	0.053 ± 0.100 (1/4)	0.055 ± 0.050 (2/4)	−
	Plot 28	0.900 ± 0.400 (4/4)	−	39.900 ± 15.300 (4/4)	0.009 ± 0.007 (0/4)	−	1.210 ± 0.520 (4/4)
Lyophilization	Plot 1	0.500 ± 0.500 (1/4)	1.400 ± 2.900 (2/4)	−	0.003 ± 0.005 (0/4)	0.003 ± 0.003 (0/4)	−
	Plot 28	0.300 ± 0.100 (0/4)	−	21.900 ± 18.700 (4/4)	0.000 ± 0.000 (0/4)	−	0.190 ± 0.070 (4/4)
Sonication	Plot 1	7.700 ± 6.800 (4/4)	8.200 ± 5.900 (4/4)	−	0.320 ± 0.240 (4/4)	5.300 ± 2.000 (4/4)	−
	Plot 28	7.100 ± 5.300 (4/4)	−	95.700 ± 5.700 (4/4)	0.670 ± 0.710 (4/4)	−	45.800 ± 8.700 (4/4)

## Data Availability

The original sequencing data generated and analyzed in this study are openly available in the NCBI GenBank database under BioProject accession number PRJNA1243456; BioSample accession numbers SAMN47622260 to SAMN47622321; and Sequence Read Archive (SRA) accession numbers SRR32908888 to SRR32908983.

## Supplementary Materials

The following supporting information can be downloaded at https://www.mdpi.com/article/10.3390/microorganisms13040923/s1, Table S1: Disease control program applied to the two apple orchards prior to the first sample collection, including details of fungicide, insecticide/miticide, and herbicide applications; Table S2: Percentage of bacterial reads (mean ± standard error, *n* = 4), targeting the V3-V4 or V4 regions of the 16S rRNA gene, retained after rarefaction and the removal of non-bacterial reads, for three pre-extraction methods (grinding, lyophilization, and sonication) across two plots (Plot 1 and Plot 28) and three time points (T1, T2, and T3). PNA blockers were not used to exclude chloroplast and mitochondrial reads. Note: ‘−’ indicates the absence of initial samples due to unavailability; Figure S1: Alpha rarefaction curve for 16S rRNA V3-V4 with PNA; 1000 reads used as cutoff to preserve maximum number of samples while still covering bacterial diversity; Figure S2: Alpha rarefaction curve for ITS; 113 reads used as cutoff to preserve maximum number of samples while still covering fungal diversity.
