# Supplementary material for: Impact of Pre-Extraction Methods on Apple Blossom Microbiome Analysis
Source: Microorganisms. 2025 Apr 16;13(4):923. doi: 10.3390/microorganisms13040923 (PMC12029539; doi:10.3390/microorganisms13040923)
Supplement: Supplementary file 1 [file microorganisms-13-00923-s001.zip › microorganisms-3516359-supplementary.pdf]

Supplementary Materials

Table S1. Disease control program applied to the two apple orchards prior to the first sample collection, including details of fungicide, insecticide/miticide, and herbicide applications.

Name	Active ingredient(s)	Chemical group	Product type	Rate	Date applied	Manufacturer
**Superior 70 Oil**	99% mineral oil	IRAC UN	Insecticide/Miticide	20L/1000 L water	March 18	Loveland Products Canada Inc., Dorchester, ON, Canada
**Folpan 80 WDG**	folpet	FRAC M4	Fungicide	3.75 kg/ha	April 20	Adama Canada Ltd., Winnipeg, MB, Canada
**Luna Tranquility**	fluopyram + pyrimethanil	FRAC 7 / FRAC 9	Fungicide	800 mL/ha	April 29	Bayer CropScience Inc., Calgary, AB, Canada
**Dithane Rainshield**	mancozeb	FRAC M3	Fungicide	1 kg/1000 L water	April 29	Corteva Agriscience™, Calgary, AB, Canada
**R/T 540 Liquid**	glyphosate	9	Herbicide	3 L/ha	May 10	Bayer CropScience Inc., Calgary, AB, Canada
**Nova**	myclobutanil	FRAC 3	Fungicide	340 g/ha	May 11	Corteva Agriscience™, Calgary, AB, Canada
**Dithane Rainshield**	mancozeb	FRAC M3	Fungicide	1 kg/1000 L water	May 11	Corteva Agriscience™, Calgary, AB, Canada

Table S2. Percentage of bacterial reads (mean ± standard error, n = 4) targeting the V3-V4 or V4 regions of the 16S rRNA gene, retained after rarefaction and the removal of non-bacterial reads, for three pre-extraction methods (grinding, lyophilization, and sonication) across two plots (Plot 1 and Plot 28) and three time points (T1, T2, and T3). PNA blockers were not used to exclude chloroplast and mitochondrial reads. Note: '-' indicates the absence of initial samples due to unavailability.

		**16S rRNA reads targeting the V3-V4 gene region (%)**			16S rRNA reads targeting the V4 region (%)		
**Method**	**Orchard**	**T1**	**T2**	**T3**	**T1**	**T2**	**T3**
Grinding	Plot 1	0.02 ± 0.02	0.02 ± 0.02	-	0.01 ± 0.01	0.01 ± 0.01	-
	Plot 28	0.00 ± 0.00	-	0.53 ± 0.36	0.00 ± 0.00	-	0.50 ± 0.24
Lyophilization	Plot 1	0.00 ± 0.00	0.00 ± 0.00	-	0.00 ± 0.00	0.00 ± 0.00	-
	Plot 28	0.00 ± 0.00	-	0.14 ± 0.11	0.00 ± 0.00	-	0.15 ± 0.13
Sonication	Plot 1	0.06 ± 0.06	0.05 ± 0.08	-	0.12 ± 0.14	0.07 ± 0.08	-
	Plot 28	0.05 ± 0.05	-	30.6 ± 20.4	0.11 ± 0.08	-	36.3 ± 21.50

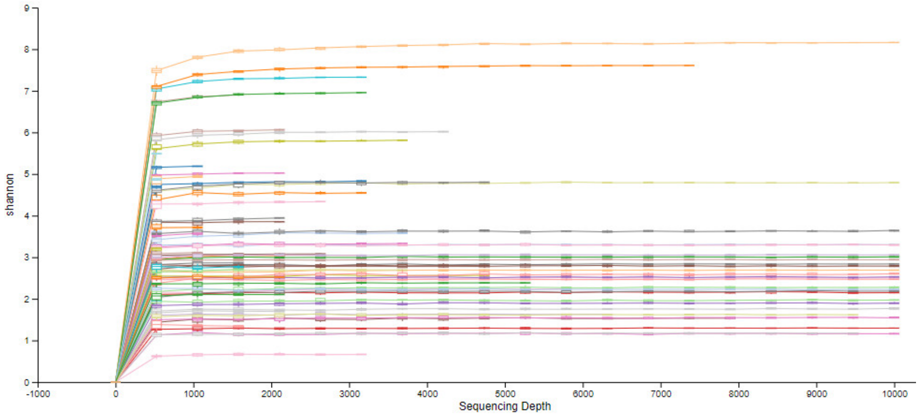

Figure S1. Alpha rarefaction curve for 16S rRNA V3-V4 with PNA; 1000 reads used as cutoff to preserve maximum number of samples while still covering bacterial diversity.

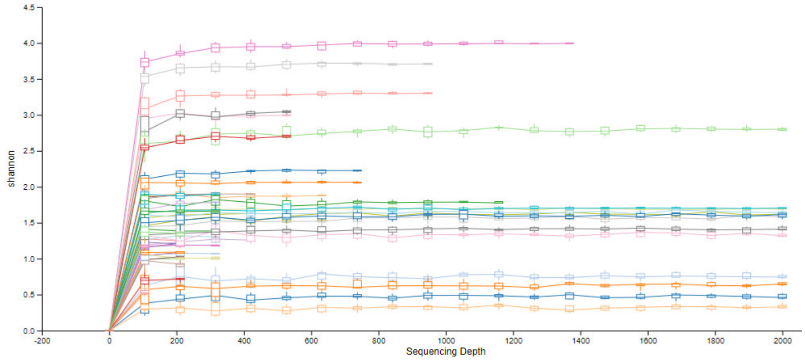

Figure S2. Alpha rarefaction curve for ITS; 113 reads used as cutoff to preserve maximum number of samples while still covering fungal diversity.

Disclaimer/Publisher's Note: The statements, opinions and data contained in all publications are solely those of the individual author(s) and contributor(s) and not of MDPI and/or the editor(s). MDPI and/or the editor(s) disclaim responsibility for any injury to people or property resulting from any ideas, methods, instructions or products referred to in the content.
